# Parathyroid Hormone Modifies the Effect of Vitamin D Supplementation on Risk of Relapse or Death in Patients with Digestive Tract Cancer: A Post Hoc Subgroup Analysis of the AMATERASU Randomized Clinical Trial

**DOI:** 10.3390/cancers18122015

**Published:** 2026-06-22

**Authors:** Akitaka Sasaki, Taisuke Akutsu, Hironori Ohdaira, Yutaka Suzuki, Ken Eto, Mitsuyoshi Urashima

**Affiliations:** 1 Division of Molecular Epidemiology, The Jikei University School of Medicine, 3-25-8, Nishi-Shimbashi, Minato-Ku, Tokyo 105-8461, Japan; akitaka.sasaki712@gmail.com (A.S.); taisuke0107.jusom@gmail.com (T.A.); 2 Department of Surgery, The Jikei University School of Medicine, 3-25-8, Nishi-Shimbashi, Minato-Ku, Tokyo 105-8461, Japan; etoken@jikei.ac.jp; 3 Department of Surgery, International University of Health and Welfare Hospital, 537-3 Iguchi, Nasushiobara, Tochigi 329-2763, Japan; ohdaira@ihwg.jp (H.O.); yutaka@ihwg.jp (Y.S.)

**Keywords:** vitamin D supplementation, parathyroid hormone, digestive tract cancer, relapse-free survival, p53, randomized clinical trial, subgroup analysis, effect modification, postoperative oncology

## Abstract

**Background/Objectives**: Parathyroid hormone (PTH), which rises compensatory with vitamin D insufficiency and has been shown to down-regulate vitamin D receptor expression, represents a biologically plausible effect modifier. We investigated whether pretreatment serum PTH modifies the effect of postoperative vitamin D supplementation on relapse-free survival, and whether adding tumor p53 status further refines subgroup identification in an exploratory analysis. **Methods**: This post hoc analysis utilized data from the AMATERASU trial (UMIN000001977), a single-center, randomized, double-blind, placebo-controlled trial evaluating vitamin D3 (2000 IU/day) versus placebo in patients with curatively resected stage I–III digestive tract cancers (maximum follow-up, 7.5 years). Patients were dichotomized at the cohort median PTH (41 pg/mL). The primary outcome was relapse-free survival (RFS), analyzed using multivariable Cox proportional hazards models. **Results**: Of 417 randomized patients, 410 had baseline PTH data. In the lower PTH subgroup (≤41 pg/mL), vitamin D significantly improved RFS compared with placebo (fully adjusted hazard ratio [HR], 0.45; 95% confidence interval [CI], 0.24–0.81; *p* = 0.008). Conversely, no benefit was observed in the higher PTH subgroup (>41 pg/mL; fully adjusted HR, 1.25; 95% CI, 0.64–2.44; *p* for interaction = 0.016). Exploratory stratification of 365 patients with p53 data showed that the supplementation benefit appeared greatest in patients with both low PTH (≤41 pg/mL) and p53-positive tumors (fully adjusted HR, 0.38; 95% CI, 0.18–0.78; *p* = 0.009). **Conclusions**: Pretreatment serum PTH is a candidate effect modifier of postoperative vitamin D supplementation in digestive tract cancers.

## Simple Summary

Vitamin D supplements may reduce cancer recurrence after surgery for digestive tract cancers, but clinical trials show inconsistent results, suggesting only certain patients benefit. We hypothesized that parathyroid hormone, which is physiologically linked to vitamin D, could predict treatment responsiveness. By reanalyzing data from a randomized trial, we found that patients with lower pretreatment parathyroid hormone levels had significantly fewer recurrences or deaths when taking vitamin D, while those with higher levels showed no benefit. Furthermore, the greatest benefit was observed in patients with both low parathyroid hormone levels and p53-positive tumors. Because parathyroid hormone is measurable by routine blood tests and p53 by standard tumor staining, combining these two markers may offer a practical approach to identifying patients most likely to benefit from postoperative vitamin D supplementation, pending prospective validation.

## 1. Introduction

Digestive tract cancers, including esophageal, gastric, and colorectal cancers, are among the leading causes of cancer-related mortality worldwide, and the risk of recurrence following curative resection remains substantial, underscoring the need for more effective adjuvant strategies [1]. Vitamin D, more precisely a precursor of the steroid hormone calcitriol, plays a critical role not only in bone and mineral metabolism but also in the regulation of diverse cellular functions relevant to oncology. Acting through the vitamin D receptor (VDR), calcitriol modulates multiple molecular pathways implicated in carcinogenesis, including inhibition of cell proliferation, induction of apoptosis and differentiation, and suppression of invasion and angiogenesis [2,3,4].

Over the past decades, numerous observational studies have suggested that higher vitamin D status is associated with improved cancer prognosis. In digestive tract malignancies in particular, prospective cohort studies have shown that higher circulating 25-hydroxyvitamin D [25(OH)D] concentrations are associated with longer survival [5,6,7]. This association has been corroborated at the meta-analytic level, with pooled analyses of cohort studies demonstrating better overall and cancer-specific survival in patients with colorectal cancer who have higher 25(OH)D [8,9], and is further supported by data showing that low plasma vitamin D predicts adverse survival after curative resection [10,11]. To test these associations in interventional settings, several large-scale randomized controlled trials (RCTs) have been conducted, although the results have been inconsistent. In the Vitamin D and Omega-3 Trial (VITAL), vitamin D3 supplementation did not significantly reduce total invasive cancer incidence, the co-primary endpoint. However, a reduction in total cancer mortality emerged in analyses that excluded the first two years of follow-up [12]. A subsequent secondary analysis of VITAL further indicated that supplementation reduced the incidence of advanced (metastatic or fatal) cancer in the overall cohort, with the strongest effect in participants of normal body weight [13]. Consistent with these findings, a meta-analysis of RCTs reported that vitamin D supplementation significantly lowered total cancer mortality despite no effect on cancer incidence [14]. In digestive tract cancer specifically, the SUNSHINE trial of high-dose versus standard-dose vitamin D3 in advanced or metastatic colorectal cancer did not meet its primary endpoint of progression-free survival, although a supportive analysis suggested a reduction in the hazard of progression or death [15].

It was within this clinical context that the primary AMATERASU trial was conducted, investigating the effect of postoperative vitamin D3 supplementation in patients with curatively resected stage I–III digestive tract cancer. In the primary analysis, supplementation did not demonstrate a statistically significant improvement in relapse-free survival (RFS) [16]. However, several post hoc subgroup analyses have suggested that the benefit of vitamin D supplementation may be concentrated in specific patient subsets. Most notably, within the AMATERASU cohort, the therapeutic advantage was highly enriched in patients with p53-positive (mutant-type) tumors [17,18], as well as in those exhibiting a prominent tumor stromal immune response [19]. Collectively, these findings highlight the clinical importance of identifying biomarkers capable of defining a biologically responsive subgroup in which the true benefit of vitamin D supplementation can be maximized.

Against this background, we focused on serum parathyroid hormone (PTH) as a candidate effect modifier. PTH is closely linked to vitamin D metabolism: as circulating 25(OH)D declines, PTH rises compensatory as part of secondary hyperparathyroidism, a relationship that is well established [20]. Pretreatment PTH may therefore serve as a physiological indicator of the vitamin D–calcium axis status at the time of surgery. Evidence linking PTH to cancer prognosis is also emerging. In pediatric cancer patients, elevated PTH—rather than low vitamin D—was the stronger predictor of inferior event-free survival [21]. However, to our knowledge, no study has examined PTH as an effect modifier of postoperative vitamin D supplementation in the context of a randomized clinical trial.

A prognostic marker is associated with outcome irrespective of treatment, whereas a predictive (effect-modifying) marker identifies patients who derive differential benefit from a specific intervention. In the present study, pretreatment PTH is evaluated specifically as a candidate predictive marker through a formal treatment-by-PTH interaction, rather than as a prognostic factor. Consistent with this, in a fully adjusted model without the interaction term, the PTH stratum carried essentially no main (prognostic) effect on relapse-free survival (HR 0.99; 95% CI, 0.64–1.54; *p* = 0.97), whereas the treatment-by-PTH interaction was statistically significant (*p* = 0.016).

We therefore conducted a post hoc subgroup analysis of the AMATERASU trial to examine whether pretreatment serum PTH modifies the effect of postoperative vitamin D supplementation on clinical outcomes in patients with digestive tract cancers (primary analysis, Analysis 1). We additionally performed an exploratory analysis examining whether the combination of pretreatment PTH and tumor p53 expression status—previously identified as an effect modifier in the AMATERASU trial—could more precisely define the patient subgroup deriving the greatest benefit from supplementation (Analysis 2).

## 2. Materials and Methods

### 2.1. Trial Design, Participants, Intervention, and Outcomes

The full design, eligibility criteria, interventions, and outcome definitions of the AMATERASU trial (UMIN000001977) have been published previously [16] and are summarized here only briefly. In short, AMATERASU was a single-center, double-blind, placebo-controlled randomized trial in which eligible adults with curatively resected stage I–III digestive tract cancer were allocated at a 3:2 ratio to postoperative oral vitamin D3 (2000 IU/day) or matching placebo [16]. The primary endpoint was relapse-free survival (RFS), defined as the time from randomization to cancer recurrence or all-cause death, whichever occurred first [16]. The study protocols were approved by the institutional review boards of International University of Health and Welfare Hospital (ethics approval code: 13-B-263; approved on 4 December 2009) and The Jikei University School of Medicine (ethics approval code: 21–216 (6094); approved on 1 January 2010) and conducted according to CONSORT guidelines; all participants provided written informed consent. Approval for the present secondary analysis was obtained separately from the same ethics boards.

### 2.2. Laboratory and Immunohistochemical Assessments

Pretreatment serum concentrations of intact PTH and 25-hydroxyvitamin D [25(OH)D] were quantified by a central laboratory (SRL Inc., Hachioji, Tokyo, Japan). In accordance with the clinical laboratory guidelines of the hospital that conducted the trial, the normal reference ranges were defined as 10–65 pg/mL for intact PTH and 8.8–10.4 mg/dL for serum calcium.

The degree of tumor p53 protein expression, based on the percentage of immunoreactive nuclear cells in the cancer glandular component, was evaluated by an investigator (T.A.) who was masked to the randomization groups and clinical outcomes [17]. A 10% cutoff point was applied for dichotomization. This threshold was selected because it is highly accurate for identifying TP53 gene status, demonstrating 84–100% sensitivity for detecting TP53 missense mutations and 86–97% specificity for the absence of such mutations [22]. Furthermore, this 10% threshold represents the most adopted cutoff point in a meta-analysis of 36 studies evaluating p53 immunohistochemistry for TP53 gene mutations, as previously validated and applied in our subgroup analysis [17,23]. Accordingly, tumors were classified into two subgroups: “p53-positive” (>10% expression), which predominantly contains missense mutations, and “p53-negative” (≤10% expression), comprising a mixture of wild-type TP53 and non-missense mutations.

### 2.3. Subgroup Definition

Of the 417 patients randomized in the AMATERASU trial, 410 had pretreatment intact PTH values available and were included in Analysis 1 (246 of 251 in the vitamin D group [98%] and 164 of 166 in the placebo group [98%]); 7 patients with missing PTH measurements were excluded. Of these 410 patients, 365 also had tumor p53 protein expression data available and were included in Analysis 2 (214 of 246 in the vitamin D group [86%] and 151 of 164 in the placebo group [92%]).

### 2.4. Statistical Analysis

Analyses followed the intention-to-treat principle. Continuous baseline variables are reported as mean ± standard deviation, and categorical variables as count and percentage, tabulated by PTH stratum and assigned treatment. The cumulative event burden within each PTH stratum over time was depicted using Nelson–Aalen hazard curves. For each subgroup comparison, treatment effects were quantified as hazard ratios (HRs) with 95% confidence intervals (CIs) derived from Cox proportional hazards models. Three hierarchically nested models were prespecified: an unadjusted model (treatment allocation only); a partially adjusted model additionally incorporating baseline 25(OH)D and serum calcium; and a fully adjusted model that further included serum creatinine, sex, age, and BMI. The selection of adjustment covariates was established a priori based on known relationships with the vitamin D–calcium–PTH axis and cancer prognosis. This three-model sequence was applied consistently across the PTH-dichotomized analysis, the four-cell PTH × p53 stratification, and a comprehensive model containing the three-way interaction term of treatment, PTH stratum, and p53 status. Adherence to the proportional hazards assumption was assessed by log–log plot inspection and scaled Schoenfeld residual tests. The global test indicated no significant violation in either stratum (*p* = 0.34 for PTH ≤ 41 pg/mL and *p* = 0.94 for PTH > 41 pg/mL), with no individual covariate showing a significant departure (all *p* > 0.10). As a sensitivity analysis, treatment effects were additionally estimated within four prespecified PTH quartiles (Q1: 8–32, Q2: 33–41, Q3: 42–53, Q4: 54–230 pg/mL) to assess the robustness of the median-based dichotomization. All computations were performed in Stata version 19.5 (StataCorp, College Station, TX, USA); two-sided *p* < 0.05 was the threshold for statistical significance. Given the post hoc and exploratory nature of this study, all findings should be interpreted with appropriate caution in the context of multiple comparisons.

## 3. Results

### 3.1. Patient Characteristics

**Figure 1:**
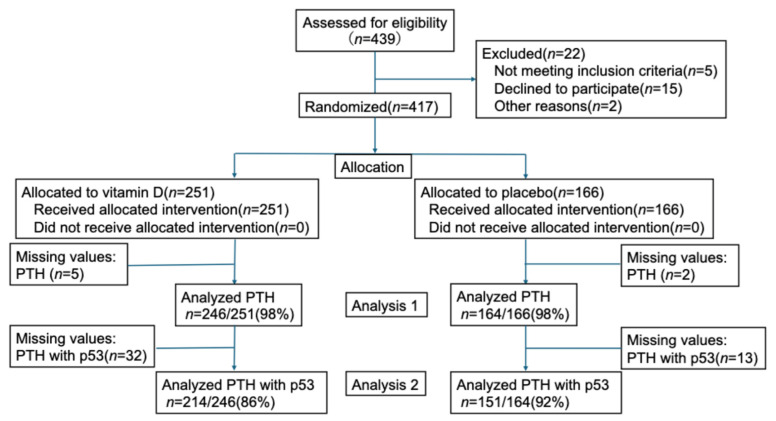
Flow diagram of patient enrolment; PTH: parathyroid hormone.

As detailed in the trial flow (Figure 1), 439 patients were initially assessed for eligibility, of whom 22 were excluded, leaving 417 patients randomized to receive either vitamin D (*n* = 251) or placebo (*n* = 166). Of the 417 patients enrolled in the AMATERASU trial, 410 had pretreatment serum PTH and 25(OH)D measurements available and were included in the present analysis. The cohort was dichotomized at the median PTH concentration of 41 pg/mL, yielding 210 patients with PTH ≤ 41 pg/mL (85 placebo and 125 vitamin D), defined as the lower PTH subgroup, and 200 patients with PTH > 41 pg/mL (79 placebo and 121 vitamin D), defined as the higher PTH subgroup. Baseline characteristics by PTH stratum and treatment assignment are shown in Table 1. The mean age was 65.8 years in the PTH ≤ 41 stratum and 66.4 years in the PTH > 41 stratum; within the PTH > 41 stratum, the placebo group was younger than the vitamin D group (62.6 vs. 68.8 years). The proportion of men ranged from 24.0% to 44.3% across the four subgroups, and the distribution of primary tumor sites was similar, with gastric cancer (40.0–42.4%) and colorectal cancer (45.6–49.6%) accounting for the majority of cases. Stage distribution and the proportion receiving adjuvant chemotherapy were also comparable across groups. Mean baseline serum 25(OH)D was lower in the PTH > 41 stratum (19.0–20.3 ng/mL) than in the PTH ≤ 41 stratum (22.9–24.0 ng/mL). Mean serum PTH was 30.6–31.1 pg/mL in the PTH ≤ 41 stratum and 58.9–63.3 pg/mL in the PTH > 41 stratum. For the analysis of PTH with p53 status (Analysis 2 in Figure 1), an additional 45 patients were excluded due to missing p53 values. To assess whether these missing p53 data were informative, we compared baseline characteristics between patients with and without available p53 data; no significant differences were observed in age, sex, BMI, pretreatment PTH, pathological stage, or adjuvant chemotherapy (all *p* > 0.05), with no evidence of systematic differences detected across the available clinicopathological variables. The proportion of patients with p53-positive tumors was 63.1–65.8% in the PTH ≤ 41 stratum, and 48.6% in the PTH > 41 placebo group versus 63.1% in the PTH > 41 vitamin D group. Given these between-group differences in age, baseline 25(OH)D, and p53 positivity, all subsequent analyses were performed using both unadjusted models and multivariable models adjusting for these and other prespecified covariates.

### 3.2. Distribution of Pretreatment Serum PTH

**Figure 2:**
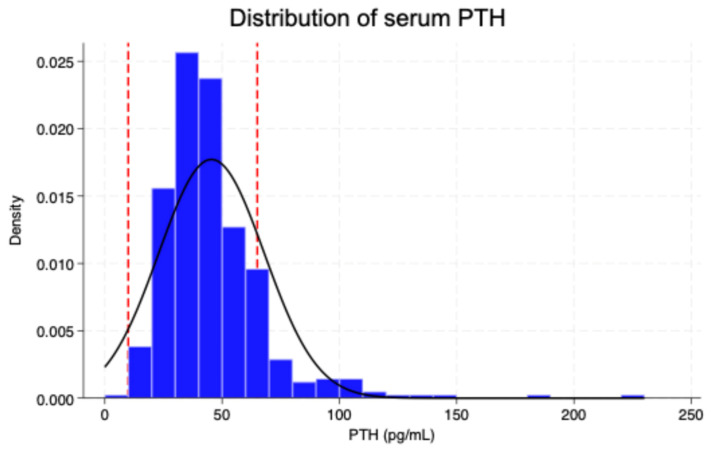
Distribution of pretreatment serum PTH among 417 randomized patients. Dashed lines indicate normal reference range (10–65 pg/mL; SRL Inc.); PTH: parathyroid hormone.

The distribution of pretreatment serum PTH among the 417 randomized patients is shown in Figure 2. PTH values were right-skewed, with a median of 41 pg/mL (interquartile range, 29–54 pg/mL) and a range of 8–230 pg/mL. A skewness-kurtosis test confirmed significant deviation from normality (Obs = 417; Pr[skewness] < 0.001; Pr[kurtosis] < 0.001; adjusted chi^2^[16] = 211.19; *p* < 0.001). Based on this non-normal distribution, the cohort median of 41 pg/mL was selected as the dichotomization threshold for subsequent analyses.

### 3.3. Effect of Vitamin D Supplementation by PTH Stratum

**Figure 3:**
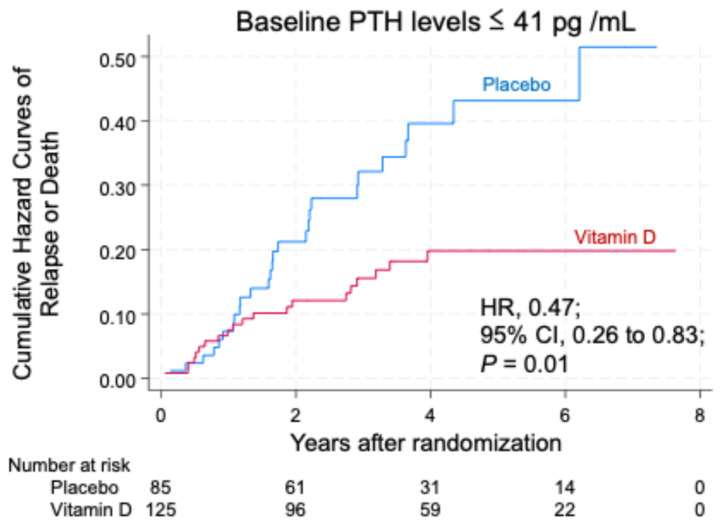
Nelson-Aalen cumulative hazard curves for relapse or death in the subgroups of PTH levels ≤ 41 pg/mL; PTH: parathyroid hormone, HR: hazard ratio, CI: confidence interval.

**Figure 4:**
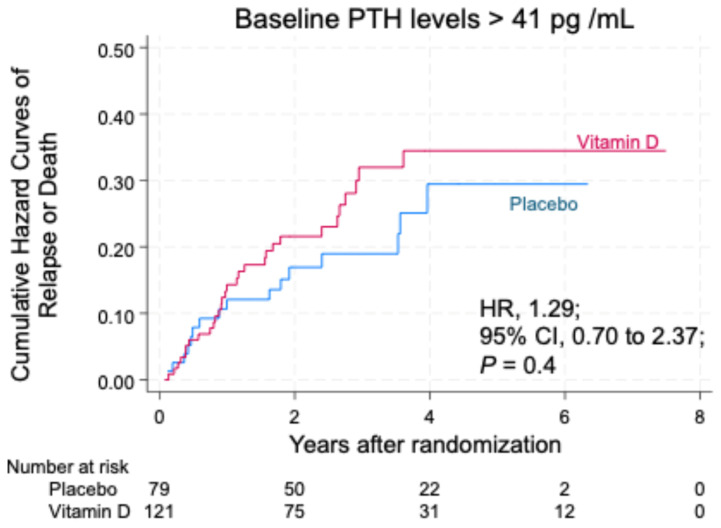
Nelson-Aalen cumulative hazard curves for relapse or death in the subgroups of PTH levels > 41 pg/mL; PTH: parathyroid hormone, HR: hazard ratio, CI: confidence interval.

During a maximum follow-up of approximately 7.5 years, a total of 93 events (relapse or death) occurred: 47 in the PTH ≤ 41 stratum (27/85 placebo, 20/125 vitamin D) and 46 in the PTH > 41 stratum (16/79 placebo, 30/121 vitamin D). Cumulative hazard curves by treatment assignment within each PTH stratum are shown in Figure 3 and Figure 4. In the PTH ≤ 41 stratum, the unadjusted hazard ratio for vitamin D supplementation versus placebo was 0.47 (95% CI, 0.26–0.84; *p* = 0.01). This estimate remained stable after partial adjustment for baseline 25(OH)D and serum calcium (HR 0.48; 95% CI, 0.27–0.86; *p* = 0.01) and after full adjustment for serum calcium, creatinine, sex, age, BMI, and baseline 25(OH)D (HR 0.45; 95% CI, 0.24–0.81; *p* = 0.008). In the PTH > 41 stratum, no significant effect of vitamin D supplementation was observed in any model (unadjusted HR 1.29; 95% CI, 0.70–2.37; fully adjusted HR 1.25; 95% CI, 0.64–2.44). The treatment-by-PTH interaction was statistically significant and stable across models (unadjusted *p* = 0.018; fully adjusted *p* = 0.016). Modeling PTH as a continuous variable yielded a consistent result (linear interaction HR 1.021 per pg/mL; 95% CI, 1.001–1.041; *p* = 0.044; restricted cubic spline joint test *p* = 0.026); Figure S1. Adding pathological stage and adjuvant chemotherapy did not alter the findings (PTH ≤ 41 stratum HR 0.45; 95% CI, 0.24–0.84; interaction *p* = 0.027). The stability of the PTH ≤ 41 estimate across the unadjusted, 25(OH)D-adjusted, and fully adjusted models indicates that the effect modification by PTH is independent of baseline 25(OH)D.

### 3.4. Sensitivity Analysis Using PTH Quartiles

**Figure 5:**
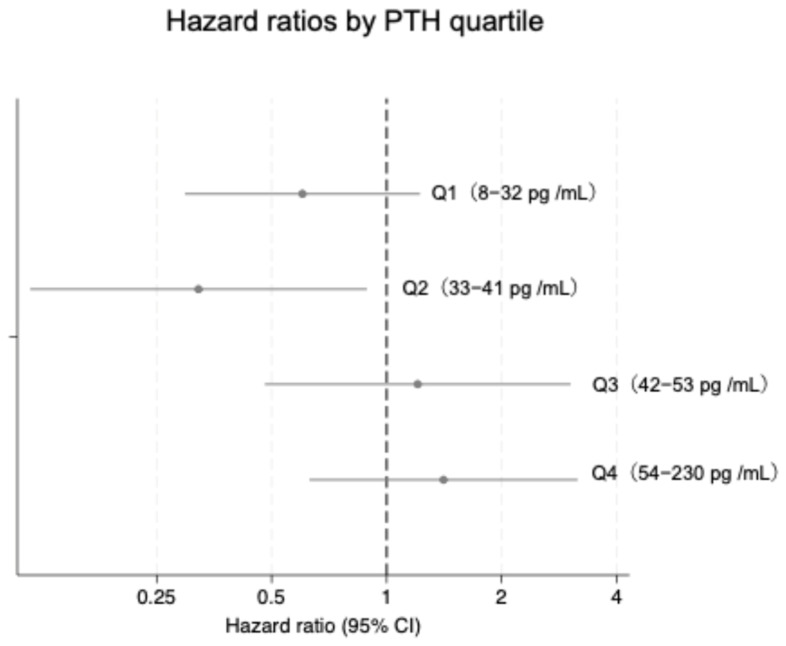
Hazard ratios for relapse or death with vitamin D supplementation versus placebo are shown for each pretreatment PTH quartile: Q1, Q2, Q3, and Q4; PTH: parathyroid hormone, HR: hazard ratio, CI: confidence interval.

To examine the robustness of the dichotomized PTH cutoff, hazard ratios of vitamin D supplementation were estimated within prespecified PTH quartiles (Q1: 8–32, Q2: 33–41, Q3: 42–53, and Q4: 54–230 pg/mL; Figure 5). The supplementation effect was concentrated in the lower two quartiles, with the largest effect observed in Q2 (33–41 pg/mL), where the 95% confidence interval excluded 1.0. In Q3 and Q4, the point estimates were close to or above 1.0, with confidence intervals crossing unity. This overall gradient across PTH quartiles was directionally consistent with the findings from the primary dichotomized analysis.

### 3.5. Joint Stratification by PTH and p53

**Figure 6:**
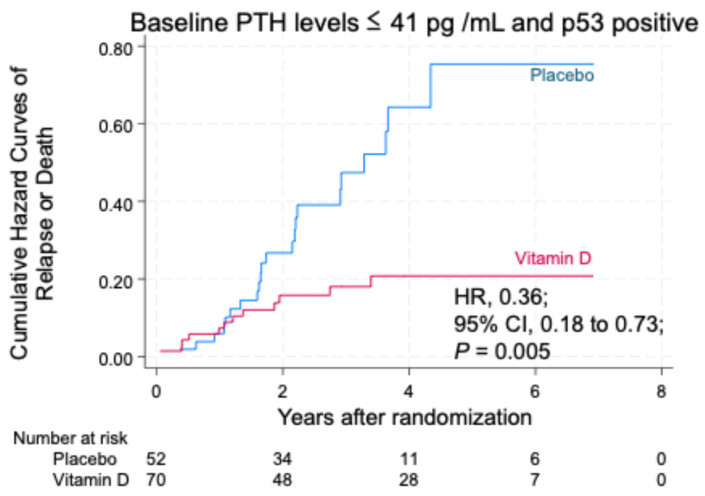
Nelson-Aalen cumulative hazard curves for relapse or death in the subgroups of PTH levels ≤ 41 pg/mL and p53 positive; PTH: parathyroid hormone, HR: hazard ratio, CI: confidence interval.

**Figure 7:**
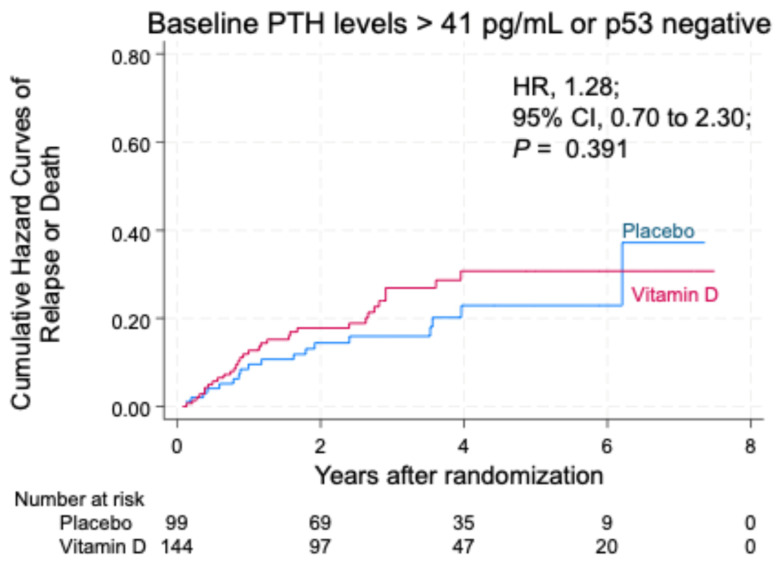
Nelson-Aalen cumulative hazard curves for relapse or death in the subgroups of PTH levels > 41 pg/mL or p53 negative; PTH: parathyroid hormone, HR: hazard ratio, CI: confidence interval.

We next examined the potential joint effect modification by tumor p53 status and PTH stratum. The absolute numbers of events (relapse or death) and patients in each of the four joint PTH × p53 subgroups are provided in Table S1. As shown in Figure 6 and Figure 7, in this exploratory analysis, the supplementation benefit appeared greatest in the subgroup of patients with both p53-positive tumors and pretreatment PTH ≤ 41 pg/mL (*n* = 122; unadjusted HR 0.36; 95% CI, 0.18–0.73; *p* = 0.005; fully adjusted HR 0.38; 95% CI, 0.18–0.78; *p* = 0.009). In the remaining three combinations of p53 and PTH status, no statistically significant effect of vitamin D supplementation was observed, with point estimates close to or above unity and confidence intervals crossing 1.0. In a combined Cox model including a three-way interaction term (treatment assignment × p53 status × PTH stratum), the hazard ratio for the p53(+) and PTH ≤ 41 pg/mL subgroup receiving vitamin D supplementation was 0.28 (95% CI, 0.11–0.70; *p* = 0.007) in the unadjusted analysis, 0.28 (95% CI, 0.11–0.69; *p* = 0.006) in the partially adjusted analysis, and 0.33 (95% CI, 0.13–0.84; *p* = 0.020) in the fully adjusted analysis.

## 4. Discussion

### 4.1. Principal Findings

This post hoc subgroup analysis of the AMATERASU randomized clinical trial demonstrated that pretreatment serum PTH is a significant effect modifier of postoperative vitamin D supplementation in patients with digestive tract cancers. Among patients with pretreatment PTH ≤ 41 pg/mL, vitamin D supplementation was associated with a substantial and statistically significant reduction in the risk of relapse or death (fully adjusted HR 0.45; 95% CI, 0.24–0.81; *p* = 0.008), with estimates remaining stable across all three hierarchically nested adjustment models. In marked contrast, no benefit was observed in patients with PTH > 41 pg/mL across any model (fully adjusted HR 1.25; 95% CI, 0.64–2.44), and the interaction between treatment assignment and PTH stratum was statistically significant (*p* for interaction = 0.016). In the exploratory joint stratification by PTH and tumor p53 status, the treatment benefit was greatest in the subgroup defined by both low pretreatment PTH and p53-positive tumors (fully adjusted HR 0.38; 95% CI, 0.18–0.78; *p* = 0.009), whereas no significant benefit was observed in the remaining three strata. These findings suggest that pretreatment PTH may act as a candidate effect modifier and that its combination with tumor p53 status may help define a more responsive patient subgroup. Given the exploratory nature and limited subgroup size, however, these observations require prospective confirmation. Because multiple subgroups were examined without formal adjustment for multiple comparisons, all subgroup-specific estimates—particularly those from the exploratory joint PTH/p53 stratification—should be regarded as hypothesis-generating.

### 4.2. Biological Rationale for the 41 pg/mL Threshold

The dichotomization threshold of 41 pg/mL used in the present analysis was the cohort median, selected a priori to avoid data-driven cutoff optimization. However, this value is also contextually interpretable from a physiological standpoint. In healthy adults with replete vitamin D status, PTH concentrations typically plateau at approximately 30 pg/mL, beyond which further increases in 25(OH)D no longer suppress PTH [20,24,25]. In contrast, a large pediatric cancer cohort reported a median PTH of approximately 47 pg/mL [21]. The cohort median of 41 pg/mL in the present study falls between these two reference points, suggesting that, as a group, patients with resected digestive tract cancer exhibit a modest upward shift in PTH relative to healthy adults—consistent with the metabolic perturbations associated with surgical stress and cancer-related vitamin D insufficiency. While the 41 pg/mL threshold has not been externally validated, it is broadly consistent with these established physiological reference points, and the directionally consistent gradient observed across PTH quartiles suggests that this value does not represent an arbitrary cutoff.

### 4.3. Mechanistic Interpretation: Why Did Vitamin D Supplementation Benefit Only the Lower PTH Subgroup?

The observation that vitamin D supplementation was effective exclusively in the low-PTH stratum is, at first glance, counterintuitive when viewed through the lens of classical calcium–vitamin D physiology. The established model proposed by Lips and colleagues holds that elevated PTH reflects tissue-level vitamin D insufficiency and secondary hyperparathyroidism [20]; under this framework, one would predict that patients with higher PTH—representing a greater degree of vitamin D deficiency—would derive the greatest benefit from supplementation. The present findings are directly contrary to this prediction, and we propose that this apparent paradox is best explained by the direct inhibitory effect of PTH on VDR expression.

Reinhardt and colleagues demonstrated in a series of in vitro and in vivo experiments that PTH is a potent down-regulator of VDR expression: treatment of osteosarcoma cells with PTH resulted in a dose- and time-dependent decline in VDR protein and a concomitant 50% reduction in VDR mRNA, and PTH co-infusion completely abolished the VDR up-regulation and downstream gene activation induced by exogenous 1,25-dihydroxyvitamin D3 in rats [26]. These findings indicate that PTH and vitamin D exert opposing effects on VDR expression, such that elevated circulating PTH may render target tissues refractory to vitamin D supplementation by suppressing the very receptor through which vitamin D mediates its antitumor effects. Under this framework, patients with pretreatment PTH > 41 pg/mL may have entered the postoperative period with constitutively suppressed VDR expression, such that exogenous vitamin D supplementation at 2000 IU/day was insufficient to overcome this receptor-level barrier and exert meaningful antitumor activity. Conversely, in patients with pretreatment PTH ≤ 41 pg/mL, VDR expression would be expected to remain intact, allowing vitamin D’s canonical antitumor mechanisms—including VDR-mediated transcriptional regulation of cell cycle arrest, apoptosis, and immune modulation—to operate at full capacity [3,27], thereby producing the substantial reduction in relapse or death observed in this stratum (fully adjusted HR 0.45). This mechanistic interpretation is consistent with the emerging concept that PTH carries independent prognostic information beyond what is captured by 25(OH)D alone, as documented in pediatric cancer patients [21] and in general population cohorts [28]. We emphasize that VDR expression, 1,25-dihydroxyvitamin D, and other mechanistic intermediates were not measured in the present study. The VDR-downregulation model is therefore offered as a biological hypothesis to be tested rather than a demonstrated mechanism.

### 4.4. Exploratory Joint Stratification by PTH and Tumor p53 Status

Building upon the primary finding of PTH as an effect modifier, the exploratory joint stratification by tumor p53 status further refined the identification of a potentially more responsive patient subgroup, with the largest treatment benefit appearing among patients with both low pretreatment PTH (≤41 pg/mL) and p53-positive tumors (fully adjusted HR 0.38; 95% CI, 0.18–0.78; *p* = 0.009).

This pattern suggests that p53 status and pretreatment PTH provide complementary and largely non-overlapping information about treatment responsiveness. Tumor p53 expression reflects a tumor-intrinsic biological state: in p53-positive tumors, which predominantly harbor missense mutations, the accumulation of dysfunctional p53 protein may render cancer cells particularly dependent on, or sensitive to, vitamin D–VDR signaling for the regulation of cell cycle progression and apoptosis [17,18,29,30,31]. Pretreatment PTH, in contrast, captures the host metabolic milieu at the time of surgery and, as elaborated in Section 4.3, may determine the functional availability of VDR through which vitamin D exerts its effects. The direct molecular interaction between p53 mutational status and PTH-mediated VDR regulation remains to be elucidated and represents an important direction for future mechanistic research. Nonetheless, the clinical evidence presented here suggests that these two biomarkers operate through mechanistically distinct and potentially complementary pathways, and that their combined assessment may help identify a candidate subgroup that could be more likely to benefit from postoperative vitamin D supplementation. Both biomarkers are assessable through routine clinical investigations—standard serum PTH measurement and immunohistochemical p53 staining—rendering this two-tier stratification approach practically implementable in prospective trial settings.

### 4.5. Limitations

This analysis has several limitations. First, it was a post hoc, exploratory investigation that was not prespecified in the original AMATERASU protocol and was not adjusted for multiple testing; the findings should therefore be regarded as hypothesis-generating. Second, sample sizes in some subgroups, particularly the p53-negative and PTH > 41 pg/mL combinations, were modest (*n* = 68 and *n* = 75, respectively), which limits the precision of the corresponding estimates. Third, the PTH cutoff of 41 pg/mL was derived from the cohort median rather than from an externally validated biological threshold, and the optimal PTH cutoff for clinical application requires prospective validation in independent cohorts. Fourth, the parent trial was conducted at a single Japanese institution, and the observed effect sizes may not translate directly to populations differing in baseline vitamin D status, dietary calcium intake, body composition, or genetic background relevant to PTH regulation. Fifth, PTH exhibits circadian variation, with concentrations typically peaking in the early morning and declining through the afternoon; however, the time of blood sampling was not recorded in the AMATERASU trial, precluding any assessment or adjustment for this source of variability, which may have introduced non-differential misclassification of the PTH stratum. Sixth, 1,25-dihydroxyvitamin D was not measured, precluding a more comprehensive assessment of the active vitamin D–PTH regulatory axis. Finally, although the multivariable models adjusted for several known confounders, residual confounding from unmeasured variables, particularly those related to bone metabolism, renal function fluctuations, and dietary factors, cannot be excluded. Prospective studies designed to evaluate vitamin D supplementation in digestive tract cancer patients stratified by pretreatment PTH and tumor p53 status, ideally with concurrent longitudinal measurements of PTH, 25(OH)D, and related analytes, are needed to confirm and extend these findings. In addition, dietary calcium intake and over-the-counter calcium supplementation were not systematically recorded in the AMATERASU trial; because calcium intake influences both PTH secretion and vitamin D–calcium homeostasis, this represents a potential unmeasured confounder that could not be adjusted for.

## 5. Conclusions

In this post hoc analysis of the AMATERASU randomized clinical trial, postoperative vitamin D supplementation was associated with a significant reduction in the risk of relapse or death, specifically in patients with pretreatment PTH ≤ 41 pg/mL, whereas no benefit was observed in patients with PTH > 41 pg/mL. Further stratification by tumor p53 status suggested that the combination of low pretreatment PTH and p53-positive tumor may identify a subgroup with greater apparent treatment benefit, supporting the concept that a two-tier stratification by host PTH status and tumor p53 expression may help generate a testable strategy for identifying patients most likely to benefit from postoperative vitamin D supplementation in digestive tract cancers. Given the exploratory and post hoc nature of this analysis, these findings should be regarded as hypothesis-generating and require confirmation in prospectively designed studies.

## Figures and Tables

**Table 1:** Baseline characteristics of patients enrolled in the AMATERASU trial by PTH stratum and treatment allocation.

	PTH ≤ 41 pg/mL		PTH > 41 pg/mL	
	Placebo (*n* = 85)	Vitamin D (*n* = 125)	Placebo (*n* = 79)	Vitamin D (*n* = 121)
Age, years	65.6 (10.3)	66.1 (10.3)	62.6 (10.2)	68.8 (10.6)
Male sex, *n* (%)	27 (31.8)	30 (24.0)	35 (44.3)	46 (38.0)
BMI, kg/m^2^	22.0 (2.8)	22.4 (3.7)	22.0 (3.1)	21.8 (3.0)
Primary tumor site, *n* (%)				
Esophageal	9 (10.6)	9 (7.2)	9 (11.4)	12 (9.9)
Gastric	34 (40.0)	53 (42.4)	33 (41.8)	50 (41.3)
Small intestinal	0 (0.0)	1 (0.8)	1 (1.3)	0 (0.0)
Colorectal	42 (49.4)	62 (49.6)	36 (45.6)	59 (48.8)
Pathological stage, *n* (%)				
Stage I	32 (37.6)	62 (49.6)	34 (43.0)	50 (41.3)
Stage II	25 (29.4)	25 (20.0)	23 (29.1)	36 (29.8)
Stage III	28 (32.9)	38 (30.4)	22 (27.8)	35 (28.9)
Adjuvant chemotherapy, *n* (%)	29 (34.1)	46 (36.8)	31 (39.2)	40 (33.1)
Serum calcium, mg/dL	9.35 (0.44)	9.31 (0.53)	9.29 (0.54)	9.19 (0.50)
Serum creatinine, mg/dL	0.74 (0.16)	0.80 (0.20)	0.74 (0.19)	0.79 (0.22)
eGFR, mL/min/1.73 m^2^	76.9 (14.6)	73.5 (16.9)	76.7 (17.2)	71.4 (16.4)
Pretreatment 25(OH)D, ng/mL	22.9 (7.2)	24.0 (8.4)	19.0 (7.9)	20.3 (7.2)
Pretreatment PTH, pg/mL	30.6 (7.1)	31.1 (7.1)	63.3 (29.5)	58.9 (17.7)
p53-positive tumor, *n* (%)	52 (65.8)	70 (63.1)	35 (48.6)	65 (63.1)

Continuous variables are presented as mean (standard deviation); categorical variables as *n* (%). BMI, body mass index; eGFR, estimated glomerular filtration rate; 25(OH)D, 25-hydroxyvitamin D; PTH, parathyroid hormone.

## Data Availability

The datasets generated and/or analyzed during the current study are not publicly available due to ethical restrictions related to patient privacy but are available from the corresponding author on reasonable request and with appropriate institutional approval.

## Supplementary Materials

The following supporting information can be downloaded at: https://www.mdpi.com/article/10.3390/cancers18122015/s1, Figure S1: Interaction of Continuous PTH on Treatment Effect; Table S1: Absolute event counts (relapse or death) by pretreatment PTH stratum and tumor p53 status.

## Abbreviations

The following abbreviations are used in this manuscript:

BMI	Body Mass Index
CI	Confidence Interval
HR	Hazard Ratio
eGFR	Estimated Glomerular Filtration Rate
ITT	Intention-to-Treat
IU	International Units
PTH	Parathyroid Hormone
RCT	Randomized Controlled Trial
RFS	Relapse-Free Survival
25(OH)D	25-Hydroxyvitamin D
