# Supplementary material for: Parathyroid Hormone Modifies the Effect of Vitamin D Supplementation on Risk of Relapse or Death in Patients with Digestive Tract Cancer: A Post Hoc Subgroup Analysis of the AMATERASU Randomized Clinical Trial
Source: Cancers (Basel). 2026 Jun 22;18(12):2015. doi: 10.3390/cancers18122015 (PMC13296973; doi:10.3390/cancers18122015)
Supplement: Supplementary file 1 [file cancers-18-02015-s001.zip › cancers-4362922-supplementary.pdf]

Supplementary Materials

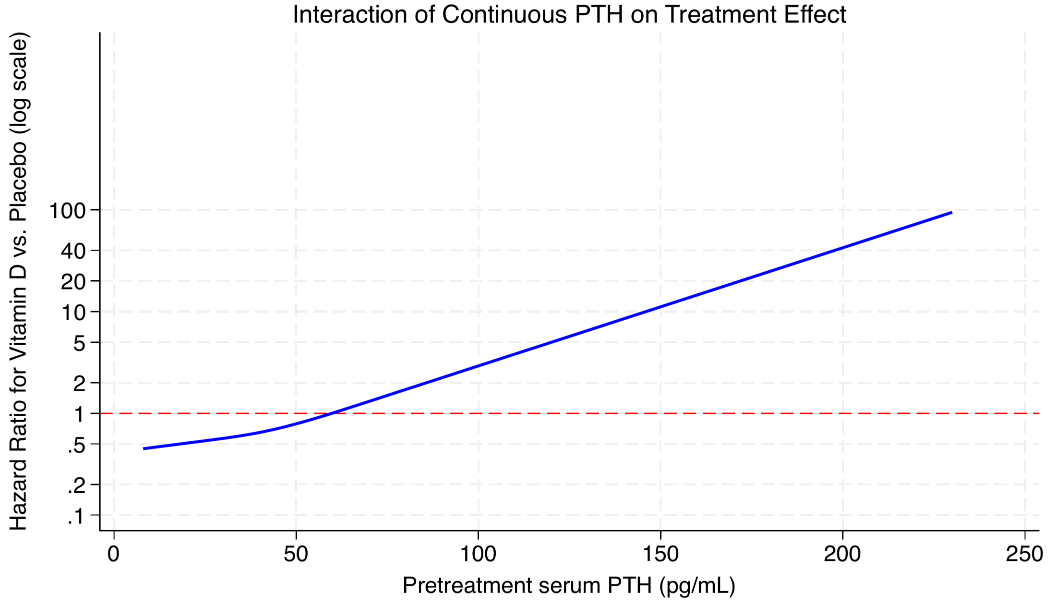

Figure S1. Treatment-by-PTH interaction modeled with pretreatment serum PTH as a continuous variable (restricted cubic spline). The solid line indicates the hazard ratio (HR, log scale) for vitamin D supplementation versus placebo across the observed range of PTH; the dashed line indicates HR = 1 (no treatment effect). The overall interaction was statistically significant (joint test, P = 0.026). HR, hazard ratio; PTH, parathyroid hormone.

Table S1. Absolute event counts (relapse or death) by pretreatment PTH stratum and tumor p53 status.

Subgroup	Placebo events (Total)	Vitamin D events (Total)
PTH ≤ 41 pg/mL & p53-positive	22 (52)	12 (70)
PTH ≤ 41 pg/mL & p53-negative	4 (27)	7 (41)
PTH > 41 pg/mL & p53-positive	8 (35)	14 (65)
PTH > 41 pg/mL & p53-negative	6 (37)	11 (38)
**Total**	**40 (151)**	**44 (214)**

Data are presented as number of events (total). The four subgroups comprise the 365 patients with available p53 data. PTH, parathyroid hormone.
